# Mechanistic Insight Into Ionizable Cationic Lipid‐Mediated Endosomal Escape via Transient Lipid Complexes

**DOI:** 10.1002/smll.202513399

**Published:** 2026-04-26

**Authors:** David Noel Zimmer, Friederike Schmid, Giovanni Settanni

**Affiliations:** ^1^ Faculty of Physics and Astronomy Ruhr University Bochum Bochum Germany; ^2^ Institute of Physics Johannes‐Gutenberg University Mainz Mainz Germany

**Keywords:** anionic/cationic lipids, endosomal escape, lamellar‐to‐hexagonal phase transition, LNP

## Abstract

RNA‐based therapeutics have demonstrated remarkable efficacy and hold great promise for future applications in personalized medicine. The most common delivery systems for these drugs are lipid‐based nanoparticles (LNPs), which incorporate ionizable cationic lipids (ICLs) as key components. Among other, ICLs are believed to facilitate endosomal escape of the cargo by interacting with anionic lipids in the endosomal membrane, although the underlying molecular mechanism remains unclear. One prevailing hypothesis suggests that membrane destabilization is mediated by cone‐shaped complexes formed between ICLs and endosomal anionic lipids. However, no clear evidence of stable co‐localization of anionic and cationic lipids has been presented so far. To address this gap, equilibrium and nonequilibrium molecular dynamics simulations of model membrane systems containing DODMA (ICL), DOPS, or PI3P (anionic lipid) and DOPE or cholesterol (helper lipid) are performed. The results confirm the absence of co‐localization at equilibrium but reveal transient formation of cone‐shaped complexes during lamellar‐to‐inverted‐hexagonal phase transitions, which considerably accelerates the transition process. These findings suggest that transient lipid–lipid interactions, rather than stable complexes, may play a critical role in facilitating endosomal escape. This mechanistic insight may inform the rational design of ICLs tailored to interact with specific endosomal anionic lipids, thereby enabling more effective and targeted delivery strategies for RNA‐based therapeutics.

## Graphical abstract

Coarse‐grained simulations show that the formation of transient anionic–cationic lipid pairs regulates the rate‐limiting step for the lamellar‐to‐hexagonal phase transition in mixtures of anionic and cationic lipids mimicking the endosomal membrane after fusion with lipid‐based nanoparticles, thus playing an important role in the endosomal escape of the LNP cargo.

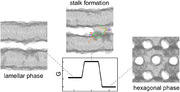

## 1 Introduction

Formulations containing cationic lipids have been studied for decades as non‐viral vectors for the delivery of nucleic acids [1, 2]. Lipid‐based nanoparticles (LNP) including ionizable cationic lipids have shown great efficacy in the COVID19 mRNA‐based vaccines [3, 4], as well as in other approved therapies [5]. Despite these advances, the detailed mechanism by which LNPs help nucleic acids transfect target cells is not yet well understood, although endocytosis is considered as the main uptake pathway [6].

Cullis and coworkers [7] demonstrated that lipid mixtures containing cationic lipids and anionic lipids, the latter present in endosomal membranes, promote the emergence of the inverted hexagonal phase over the lamellar phase. The same authors proposed that this mechanism underlies the destabilization of the endosomal membrane upon LNP fusion, thereby enabling endosomal escape of the cargo [7]. Recent studies have corroborated that lamellar LNPs undergo a transition to the inverse hexagonal phase upon interaction with anionic membranes [8], and that LNPs with pre‐programmed hexagonal phases exhibit enhanced delivery efficiency. In addition, it was shown that the pH‐dependent transition between lyotropic mesophases including the inverse‐hexagonal phase was a key determinant of the efficacy of ICL formulations [9, 10], and can be tuned by buffer composition [11].

Super‐resolution microscopy has further clarified that protonated ICLs localize at the early endosomal membrane near the LNP contact site, inducing membrane disruption and facilitating RNA escape [12], although the specific molecular mechanism remains unclear. Aliakbarinodehi et al. [13] showed that LNPs including the cationic lipid MC3‐DMA were able to fuse to supported lipid bilayers mimicking endosomal membranes (that is including anionic and zwitterionic lipids) only under acidic conditions (pH≤6.0), resulting in partial RNA release. The role of anionic lipids in favoring transfection was further evidenced by Chen et al. [14] who showed that incorporation of anionic lipids such as PS or PA to the standard 4‐component LNP formulations increased their transfection efficiency.

Collectively, these data support the idea that endosomal escape involves a (localized) destabilization of the endosomal membrane governed by a lamellar to inverse‐hexagonal phase transition facilitated by anionic–cationic lipids interactions. Nevertheless, other mechanisms have also been suggested [15] and the organization of the RNA cargo within the LNPs, which depends on loading level, has been shown to influence delivery outcomes [16, 17].

The molecular determinants leading to a stabilization of the hexagonal over the lamellar phase in mixture of cationic and anionic lipids has not been fully clarified, yet. A hypothesis has been formulated [7] that cationic–anionic lipid ion pairs may give rise to cone‐shaped complexes via electrostatic headgroup interactions. According to the theory of self‐assembly of amphiphiles [18], inverted cone‐shaped moieties (with a critical packing parameter larger than 1), tend to favor the inverted hexagonal phase. However, recent molecular dynamics (MD) simulation data reporting the spatial distribution of a mixture of anionic and ionizable cationic lipids assembled in the inverted hexagonal phase, did not show co‐localization of anionic and ionizable cationic lipids [19], apparently in contrast with the cone‐shaped hypothesis.

MD simulations have helped shed light on the mechanisms involved in the delivery of nucleic acids by lipid‐based nanoparticles [20, 21, 22, 23, 24, 25, 26, 27, 28, 29, 30]. In particular, Bruininks et al. [31] showed, using a coarse‐grained model, how small segments of double‐stranded DNA could be transfected through a model endosomal membrane by a model LNP. Similarly, Kjølby et al. [32], after developing parameters for a large set of ionizable cationic lipids compatible with the MARTINI 3 coarse‐grained force field [33], provided protocols to build the structures of LNPs and test the fusion properties of formulations. Coarse‐grained simulations of lipids mixtures have qualitatively recapitulated the experimentally observed phase diagram capturing the emergence of hexagonal phase at elevated temperature and low hydration level and lamellar phase at lower temperature and high hydration level [34].

Here, using coarse‐grained molecular dynamics simulations, we study the lamellar to inverted‐hexagonal phase transition of lipid formulations, including anionic and/or cationic lipids mimicking the lipid mixing following fusion of LNPs with the endosomal membrane. The choice of the model systems is based on several considerations including earlier experiments [7] showing destabilization of the lamellar phase upon mixing of cationic and anionic lipids, as well as the anionic lipid content of early endosomal membranes [35, 36], an enrichment of cationic lipids in the endosomal membrane at the contact point with LNPs [12] and a possible neutralization of the membrane following a LNP fusion event [37], as it will be explained later in more detail. The variability of the results to the exact choice of the model systems has been evaluated by varying the lipid composition in terms of helper lipids, anionic lipids and relative abundance of anionic and cationic lipids. We first build a simple phase diagram as a function of temperature and water content for the different lipid mixtures, showing that the presence of both cationic and anionic lipids promotes the emergence of non‐lamellar and hexagonal phases relative to mixtures containing only one charged species. Then, we analyze the spatial distribution of lipids in the various phases and ascertain the absence of a clear co‐localization of cationic and anionic lipids. Finally, we analyze the actual transition events from lamellar to non‐lamellar phases. The main finding of this work is the observation of a transient co‐localization of anionic and cationic lipids initiating the phase transition and, thus, determining its speed.

## 2 Results and Discussion

Several molecular systems were built using a coarse‐grained representation of the lipids based on the MARTINI 2 [38, 39, 40] force field with the MARTINI polarizable water model [41]. Although a more accurate coarse‐grained representation of the lipids has been recently proposed within the MARTINI 3 framework [32, 33, 42], here we have preferred to use a representation that allows for a more accurate treatment of the long‐range electrostatic interactions, which play a fundamental role in the present systems. To highlight the effect of anionic/cationic lipid mixing, we considered the following formulations: (a) mixtures of anionic and helper lipids in 50/50 molar ratio; (b) mixtures of ionizable cationic and helper lipids in 50/50 molar ratio; (c) mixtures of anionic, ionizable cationic, and helper lipids with molar ratios of 25/25/50 and 12.5/37.5/50, respectively, which help explore the effect of charge balance in the mixture.

The lipids 1,2‐dioctadecenoyl‐sn‐glycero‐3‐phosphoserine (DOPS) and phosphatidylinositol 3‐phosphate (PI3P) were selected as anionic lipid as both of them are enriched in early endosome [36], where successful escape is more probable [43]. According to data from Van Meer et al. [44] and Leventis and Grinstein [45] the early endosomal membrane may include 10%–16% of anionic (PS and PI) lipids. LNPs proximity, however, is assumed to change this balance locally due to electrostatic interactions increasing the local fraction of anionic lipids in the vicinity of the fusion point. A similar migration and exposure of ionized cationic lipids on the surface of LNPs in the vicinity of the fusion point can also be assumed. These assumptions were put forward in the interpretation of experiments on the fusion of LNPs with endosomal membrane mimics, which showed that a delay is observed between the pH‐induced protonation of the ionizable lipids and the fusion of the LNP and that the delay is significantly increased by the addition to the LNP of gel‐phase forming DSPC lipids, which reduce lipid diffusion [13, 46]. Upon fusion, the LNP releases the ionized lipids into the endosomal membrane at the point of contact. The mixing leads to a local charge neutralization as evidenced by experiments showing a significant reduction in LNP fusion events on endosomal membrane mimics in the proximity of previous LNP fusion locations [37]. Thus, it is reasonable to assume that locally at the LNP fusion point a good fraction of the lipids are anionic and cationic in an amount, which leads to charge neutralization, and that the fraction of anionic lipids can be larger than the one observed on average in early endosomal membranes (10%–16%).

The lipid 1,2‐dioleyloxy‐3‐dimethylaminopropane (DODMA) in its protonated form was selected as ionizable cationic lipid. The latter, although not used in present‐day commercial LNPs, was selected because it is still considered a well‐established benchmark and because, due to its simple structure, it has been already modeled and validated within the same coarse‐grained force‐field described above [26]. The protonation state of PI3P was chosen to be −3e to mimic early endosomal membrane. DODMA was considered fully protonated based on several considerations: (1) the ${\mathrm{pK}}_{a}$ of water‐soluble analogous of DODMA is 8.93 while the experimentally determined apparent ${\mathrm{pK}}_{a}$ of DODMA‐based LNP formulations is 6.59 and titration curves show that at pH 6.0 almost 80% of DODMA lipids are protonated [47]; (2) the lipids that we are simulating are those that underwent fusion with the endosomal membrane, thus they originate from the surface of the LNP, and, being the most exposed to the acidic endosomal environment, represent the fraction of DODMA lipids of the formulation with the largest probability to be protonated even in early endosome, while the DODMA lipids in the interior of the LNP are more protected from the acidic environment and are more likely neutral. Although experimental data support the idea of a local neutralization of the membrane in the vicinity of the fusion point [37], in the case of mixed formulations including PI3P, we considered two cases: PI3P25/DODMA/DOPE with molar fractions of 25/25/50, respectively, which requires the addition of neutralizing counterions, and PI3P12/DODMA/DOPE with molar fractions 12.5/37.5/50, respectively, where DODMA lipids are sufficient to neutralize the PI3P lipids.

The lipids 1,2‐dioleoyl‐sn‐glycero‐3‐phosphoethanolamine (DOPE) and cholesterol were selected as helper lipids. Details about the force field parameters adopted for the different molecules are provided in the Methods section and a list of the simulated formulations is reported in Table 1. The coarse‐grained models of the lipids chosen here provide a rather general representation of the system aimed to cover a broad spectrum of specific lipid mixtures.

**Table 1:** List of the simulated systems, including lipid types, molar fraction, amount of water per lipid, total number of particles and box size.

Name	Formulation	No. lipids (anionic/cationic/helper)	w/l	No. particles	Initial box size x,z [nm]
Single bilayer					
PS_PE_1	DOPS/DOPE	256/0/256	1	7708	11.33, 7.36
PS_PE_2	DOPS/DOPE	256/0/256	2	9688	12.25, 5.15
PS_PE_3	DOPS/DOPE	256/0/256	3	10912	11.78, 8.26
PS_PE_4	DOPS/DOPE	256/0/256	4	12976	12.44, 5.76
DMA_PE_1	DODMA/DOPE	0/256/256	1	7455	12.06, 4.68
DMA_PE_2	DODMA/DOPE	0/256/256	2	9432	12.17, 5.08
DMA_PE_3	DODMA/DOPE	0/256/256	3	10644	12.15, 5.39
DMA_PE_4	DODMA/DOPE	0/256/256	4	12720	12.40, 5.67
PS_DMA_PE_1	DOPS/DODMA/DOPE	128/128/256	1	7582	11.65, 4.97
PS_DMA_PE_2	DOPS/DODMA/DOPE	128/128/256	2	9304	12.06, 5.06
PS_DMA_PE_3	DOPS/DODMA/DOPE	128/128/256	3	10552	12.16, 5.29
PS_DMA_PE_4	DOPS/DODMA/DOPE	128/128/256	4	12976	11.99, 5.87
PS_CHL_1	DOPS/CHOL	256/0/256	1	7708	11.33, 7.36
PS_CHL_2	DOPS/CHOL	256/0/256	2	9688	12.25, 5.15
PS_CHL_3	DOPS/CHOL	256/0/256	3	10912	11.78, 8.26
PS_CHL_4	DOPS/CHOL	256/0/256	4	12976	12.44, 5.76
DMA_CHL_1	DODMA/CHOL	0/256/256	1	7455	12.06, 4.68
DMA_CHL_2	DODMA/CHOL	0/256/256	2	9432	12.17, 5.08
DMA_CHL_3	DODMA/CHOL	0/256/256	3	10644	12.15, 5.39
DMA_CHL_4	DODMA/CHOL	0/256/256	4	12720	12.40, 5.67
PS_DMA_CHL_1	DOPS/DODMA/CHOL	128/128/256	1	7582	11.65, 4.97
PS_DMA_CHL_2	DOPS/DODMA/CHOL	128/128/256	2	9304	12.06, 5.06
PS_DMA_CHL_3	DOPS/DODMA/CHOL	128/128/256	3	10552	12.16, 5.29
PS_DMA_CHL_4	DOPS/DODMA/CHOL	128/128/256	4	12976	11.99, 5.87
PI_PE_1	PI3P/DOPE	256/0/256	1	9472	12.30, 5.52
PI_PE_2	PI3P/DOPE	256/0/256	2	11008	12.60, 5.63
PI_PE_3	PI3P/DOPE	256/0/256	3	12460	12.68, 5.92
PI_PE_4	PI3P/DOPE	256/0/256	4	14080	12.79, 6.18
PI12_DMA_PE_1	PI3P/DODMA/DOPE	64/192/256	1	7744	12.09, 4.67
PI12_DMA_PE_2	PI3P/DODMA/DOPE	64/192/256	2	9280	12.21, 5.01
PI12_DMA_PE_3	PI3P/DODMA/DOPE	64/192/256	3	10816	12.30, 5.20
PI12_DMA_PE_4	PI3P/DODMA/DOPE	64/192/256	4	12352	12.58, 5.38
PI25_DMA_PE_1	PI3P/DODMA/DOPE	128/128/256	1	8320	11.97, 5.13
PI25_DMA_PE_2	PI3P/DODMA/DOPE	128/128/256	2	9856	12.227, 5.31
PI25_DMA_PE_3	PI3P/DODMA/DOPE	128/128/256	3	11392	12.44, 5.48
PI25_DMA_PE_4	PI3P/DODMA/DOPE	128/128/256	4	12928	12.55, 5.75
Double bilayer					
PS_PE_D	DOPS/DOPE	512/0/512	2	19376	12.43, 9.99
DMA_PE_D	DODMA/DOPE	0/512/512	2	18864	12.15, 10.11
PS_DMA_PE_D	DOPS/DODMA/DOPE	256/256/512	2	18104	12.57, 9.49
PI_PE_D	PI3P/DOPE	512/0/512	2	22016	12.88, 10.86
PI12_DMA_PE_D	PI3P/DODMA/DOPE	128/384/512	2	18560	12.08, 10.34
PI25_DMA_PE_D	PI3P/DODMA/DOPE	256/256/512	2	19712	12.54, 10.22

All the formulations were initially set up to form a bilayer with a random distribution of lipids in each leaflet using the program INSANE [48]. To study the phase diagram of each of the three listed formulations, simulations were performed varying the temperature of the system (the values of 270, 285, 300, and 315 K were considered) and the hydration level (values of approximately 1, 2, 3 and 4 polarizable MARTINI [41, 49] water beads per lipid (w/l) were considered, equivalent to 4, 8, 12 and 16 water molecules per lipid, respectively).

The choice of a range of temperatures and hydration levels rather than a single value is dictated by the fact that the transition temperature may vary depending on lipid mixture and hydration level, and experimental lipid mixture transition behaviors are difficult to reproduce exactly with molecular dynamics simulations [42]. Thus, by exploring multiple points in phase space, we can more reliably compare the stability of the different lipid formulations. In particular, the lamellar‐to‐hexagonal transition temperature of a mixture of DPPS and DODMA was located between 30
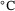
–35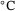 [50], thus within the range of temperatures that we are exploring.

Neutralizing ions were added to the systems. We note that, given the small amount of water beads present in the systems, imposing physiological ion concentration would require to include a very small amount of ions (6–22 of each polarity), largely outnumbered by the neutralizing ions in most of the systems. The systems were made periodic in all directions. After an initial minimization and equilibration of the systems, the production runs of 3 μs were performed at constant pressure (1 atm and semi‐isotropic pressure coupling) and temperature using the program GROMACS 2020 [51, 52, 53, 54] and replicated four times to increase statistical accuracy.

The non‐lamellar phases observed at the end of some of the simulations may show also distorted hexagonal phases, where the water columns have an elliptical base as well as intermediate phases where the parallel water columns are not fully separated but are connected by water bridges (Figure S1). Since this behavior could be induced by the semi‐isotropic pressure coupling, the simulations showing distorted hexagonal or intermediate phases were continued with an anisotropic pressure coupling for 3–12 further μs (see Methods section for details). Some of those simulations were carried out using the program OpenMM [55]. With this approach, the distorted hexagonal phases and some of the intermediate phases relaxed to regular hexagonal phases. The technical details of the simulation protocol are reported in the Methods section.

### 2.1 Destabilization of Lamellar Phase by Mixing Cationic and Anionic Lipids

The simulations described above starting from a single bilayer setup converge in less than 0.3 μs, according to the time series of the tail order parameters (Figure S2), with the exception of those including cholesterol and undergoing a phase transition (Figure S3). The slower convergence in cholesterol simulations is related to the lipid diffusion along the membrane, which is much lower than in DOPE formulations: a 2D mean‐squared‐displacement analysis shows that the diffusion coefficient of lipid heads in systems at 300K including cholesterol is 0.65±0.14 ${\mathrm{nm}}^{2}$/μs compared to 15.4±1.1 ${\mathrm{nm}}^{2}$/μs in DOPE formulations. Thus, while a structural analysis of cholesterol simulations is possible, and will be presented below along with the other formulations, the analysis of the kinetics would require a very large computational effort out of the scope of this work.

**Figure 1:**
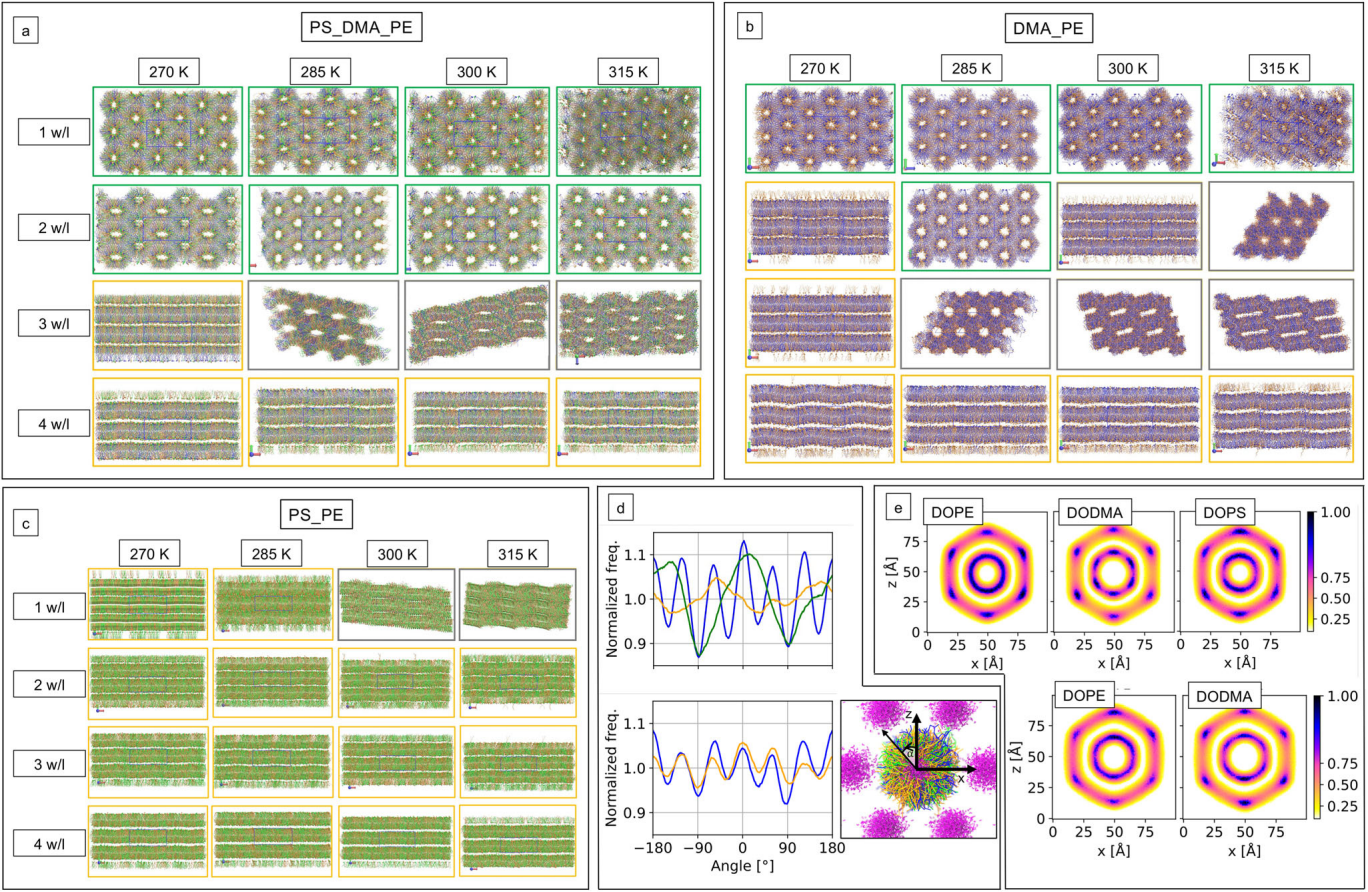
Equilibrium structures of the simulations of DOPS/DODMA/DOPE (a), DODMA/DOPE (b), and DOPS/DOPE (c). DODMA is shown in blue, DOPS in green and DOPE in orange. Water was removed for clarity, the lipids are visualized using the licorice representation in VMD. The blue parallelograms represent the boundary of the periodic box. The green and grey frames around the pictures indicate simulations that have been extended using anisotropic pressure coupling using GROMACS or OpenMM, respectively. All replicate runs end up in the same phase. Snapshots from simulation set 1. (d) Normalized angular frequency of observation of DOPS (green), DODMA (blue) and DOPE (orange) from the DOPS/DODMA/DOPE formulation (top) and from the DODMA/DOPE formulation (bottom) around the water column in the hexagonal phase. Diagram showing the angle definition (bottom right), water is shown in magenta. (e) Two‐dimensional histograms of the xz‐distribution of the charged head beads (inner ring) and the terminal tail beads (outer ring) of the indicated lipids in the DOPS/DODMA/DOPE formulation (top) and in the DODMA/DOPE formulation (bottom). The data in (d) and (e) are averaged (where possible) over the last 1 μs and over replicate runs of simulation set 3 (see Methods) at 315 K. Similar patterns are observed in the other simulations where the hexagonal phase is observed.

**Figure 2:**
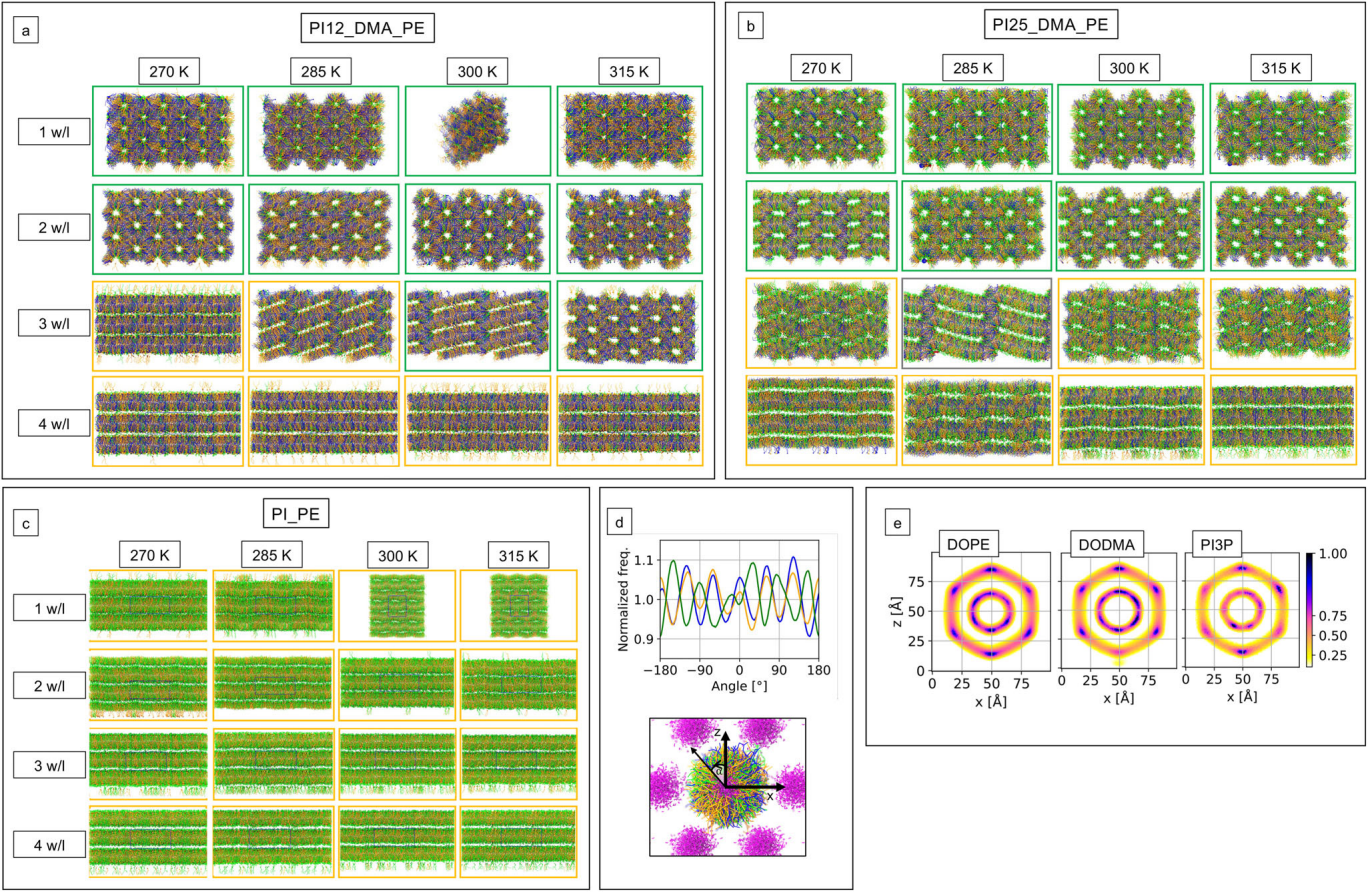
Equilibrium structures of the simulations of PI3P12/DODMA/DOPE (a), PI3P25/DODMA/DOPE (b), and PI3P/DOPE (c). DODMA is shown in blue, PI3P in green and DOPE in orange. Water was removed for clarity, the lipids are visualized using the licorice representation in VMD. The blue parallelograms represent the boundary of the periodic box. The green and gray frames around the pictures indicate simulations that have been extended using anisotropic pressure coupling using GROMACS or OpenMM, respectively. All replicate runs end up in the same phase. Snapshots from simulation set 1. (d) Normalized angular frequency of observation of PI3P (green), DODMA (blue), and DOPE (orange) from the PI3P12/DODMA/DOPE formulation around the water column formed in the hexagonal phase. Scheme of the angle definition (bottom). (e) Two‐dimensional histograms of the xz‐distribution of the charged head beads (inner ring) and the terminal tail beads (outer ring) of the indicated lipids in the PI3P12/DODMA/DOPE formulation. The data in (d) and (e) are averaged (where possible) over the last 1 μs and over replicate runs of simulation set 3 (see Methods) at 315 K. Similar patterns are observed in the other simulations where the hexagonal phase is observed.

The molecular configurations observed at the end of the simulations (Figures 1, 2; Figure S4a–c) show that in all systems including only anionic lipids (Figures 1, 2, and S4c), the lamellar phase is observed under most of the simulated conditions and replicates, with the exception of two conditions (three for DOPS/CHOL) at the smallest hydration level, where non‐lamellar phases are observed. Preference for lamellar phases is associated with a critical packing parameter close to 1, corresponding to a cylindrical shape of the lipids [18].

In the case of cationic lipid mixtures, DODMA/DOPE and DODMA/CHOL, non‐lamellar phases emerge at the lowest hydration levels, depending on temperature (Figures 1b and S4b).

The simulations of systems, including mixtures of anionic and cationic lipids, show that non‐lamellar phases emerge in a larger set of hydration and temperature conditions than the other systems (Figures 1, 2, and S4). This is in agreement with the early experimental finding by Cullis and coworkers [7] showing the destabilization of the lamellar phase by mixing cationic and anionic lipids.

The emergence of non‐lamellar phases is observed also in the system PI3P25/DODMA/DOPE where the charge of the cationic lipids does not compensate the one of the anionic lipids and sodium ions need to be introduced to neutralize the system. This indicates that ions, although normally reaching quite close to the phosphate groups (Figures S5–S7) and possibly playing a role in the determination of the effective head size of anionic lipids, as revealed by an effect of their presence on the average area per lipid (Table S1), do not interfere significantly with the lamellar/hexagonal phase equilibrium.

### 2.2 Lack of Co‐Localization of Cationic and Anionic Lipids in the Hexagonal Phase

To verify the presence of cone‐shaped complexes formed by pairs of cationic and anionic lipids in the ternary mixtures, an approach proposed by Ramezanpour et al. [19] was adopted where the density of lipids around the water columns present in the hexagonal phase was averaged along the trajectories. The angular density distributions show well‐defined patterns (Figures 1, 2; Figure S8): DODMA head bead angular density shows six evenly spaced peaks and six valleys, the latter occurring at the angles pointing toward the centers of neighbor columns in the hexagonal lattice. DOPE shows a distribution very similar to the one of DODMA in the DODMA/DOPE and PI3P12/DODMA/DOPE formulations while it is less regular in DOPS/DODMA/DOPE and DOPS/DODMA/CHOL formulations. DOPS angular distributions are less regular than the other lipids in the DOPS/DODMA/DOPE formulation, generally showing a lower number of peaks and valleys, while in the DOPS/DODMA/CHOL formulations more than six peaks are observed and they do not match DODMA peaks. Similarly, PI3P in the PI3P12/DODMA/DOPE, shows density peaks not matching DODMA density peaks.

The regular distribution of DODMA around the columns may originate from the repulsive electrostatic interactions from the DODMA lipids around neighbor columns, although the same effect is not observed for DOPS or PI3P. DOPS shows two broader peaks and two valleys, possibly related to its preference for the lamellar phase (Figure 1c), while PI3P density peaks alternate with DODMA peaks. A steric origin for the distributions of DOPS, PI3P, DODMA, and DOPE is also possible, as also shown by the hexagonal distribution of the terminal tail beads (Figure 1e) accumulating in the regions most distant from the water columns, although it does not explain the lack of co‐localization of anionic and cationic lipids. A similar steric origin for the distribution of cholesterol in formulations including anionic and cationic lipids in hexagonal phase was also observed in ref.  [19].

A more detailed analysis of Figures 1e and 2e shows that DODMA charged head bead tends to be partly buried under the phosphate group of surrounding phospholipids, a phenomenon already observed in atomistic simulations of bilayers including DODMA [26]. The inward shift of DODMA implies the need to pack a longer string of tail beads than the other lipids in the intercolumnar space, which may lead to a preference for the region equidistant from neighbor columns, offering a larger volume.

The data, most importantly, do not show a clear angular co‐localization of anionic and cationic lipids in the hexagonal phase, as expected in the case of the formation of cone‐shaped complexes. This result is in agreement with similar findings reported in ref.  [19] using atomistic simulations of a lipid mixture containing cationic and anionic lipids in the hexagonal phase.

### 2.3 Mixing Cationic and Anionic Lipids Lowers the Lamellar‐to‐Non‐Lamellar Phase Free Energy Barrier

Although anionic–cationic lipid co‐localization was not clearly observed in the equilibrated hexagonal lipid assemblies, we investigated the possibility of the emergence of anionic–cationic lipid cone‐shaped complexes as transient structures playing a role in the transition from lamellar to non‐lamellar phases. The simulations used for the phase diagrams shown above, which were started from single bilayer conformations, did not allow to properly sample the kinetics of the lamellar to non‐lamellar transition because the formation of fusion stalks between the opposite leaflets started already during the restrained equilibration phase.

To sample the kinetics of the transition we then defined larger systems containing two stacked bilayers, obtained by duplicating along the *Z* direction the configurations obtained from the previous set of simulations at the lowest temperature (270 K) and at a hydration level of 2 water beads per lipid (for both the DOPS/DODMA/DOPE and PI3Px/DODMA/DOPE systems the hydration level of 3 w/l was used and water molecules were gradually removed to reach w/l = 2, see the Methods section for details). The systems were then simulated at the temperatures of 300 and 315 K, with 10 replicate runs for each system and temperature. In these new sets of simulations, the appearance of stalks between opposite bilayers (see the Methods section for a detailed definition and validation) is used as a marker for the initiation of the transition from lamellar to non‐lamellar phases. The time point where the first stalk forms is collected for each of the simulations where a transition occurs.

**Figure 3:**
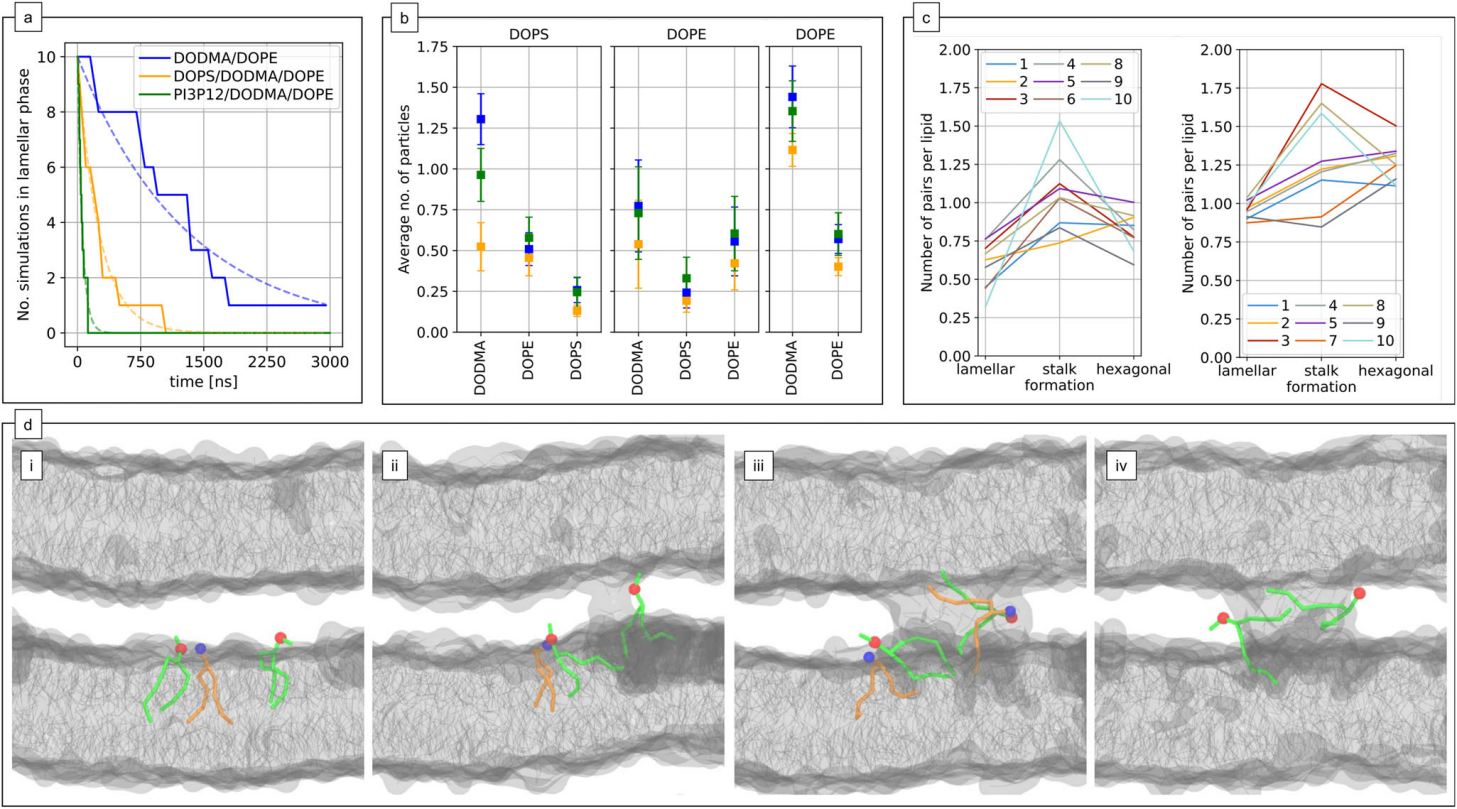
(a) Time series of the number of simulations in the lamellar phase for DOPS/DODMA/DOPE systems (orange), DODMA/DOPE systems (blue) and PI3P12/DODMA/DOPE (green). Exponential decays f(x)=10·exp(−x/τ) fitting the data are reported. Data from simulation set 2 at 315 K. (b) Average number of charged head beads ($Q_{d}$ for DODMA, $Q_{a}$ for DOPS and DOPE) of the type reported on the X axis around the heads of stalk‐initiating lipids in the DOPS/DODMA/DOPE (left for DOPS, center for DOPE), and in the DODMA/DOPE formulation (right for DOPE) at 315 K and 2 w/l. Data from simulation set 2. The data is averaged over 20 ns windows in the lamellar phase (orange), during the stalk‐formation event (blue), and in the hexagonal phase (green), the standard deviation over the replicate runs is given as error bars. (c): Average number of DODMA lipids in contact with stalk‐initiating lipids in the DOPS/DODMA/DOPE (left, data for stalk‐initiating DOPS) and DODMA/DOPE (right, data for stalk‐initiating DOPE) formulation as a function of the phase of the simulations. The stalk‐initiating lipids were identified at the stalk‐formation event, then, the same lipids were used to compute the number of contacting molecules in the lamellar and hexagonal phase, that is at the beginning and at the end of the simulation. (d) Snapshots of the stalk‐formation process in replicate run 1 of the DOPS/DODMA/DOPE formulation at 315 K and 2 w/l. The carbon chains of all lipids are shown as gray thin lines and as enveloping surface. Two stalk‐initiating DOPS molecules are shown in green with the phosphate group in red. DODMA molecules closer than 6.5 Å from the highlighted DOPS lipids are shown in orange with the methylamine group in blue. Water and non‐tail beads of all the other lipids are hidden for clarity. Snapshots are shown at (i) 255.0 ns, (ii) 259.1 ns, (iii) 264.1 ns, and (iv) 270.3 ns (the stalk‐formation event starts at 253.6 ns).

**Figure 4:**
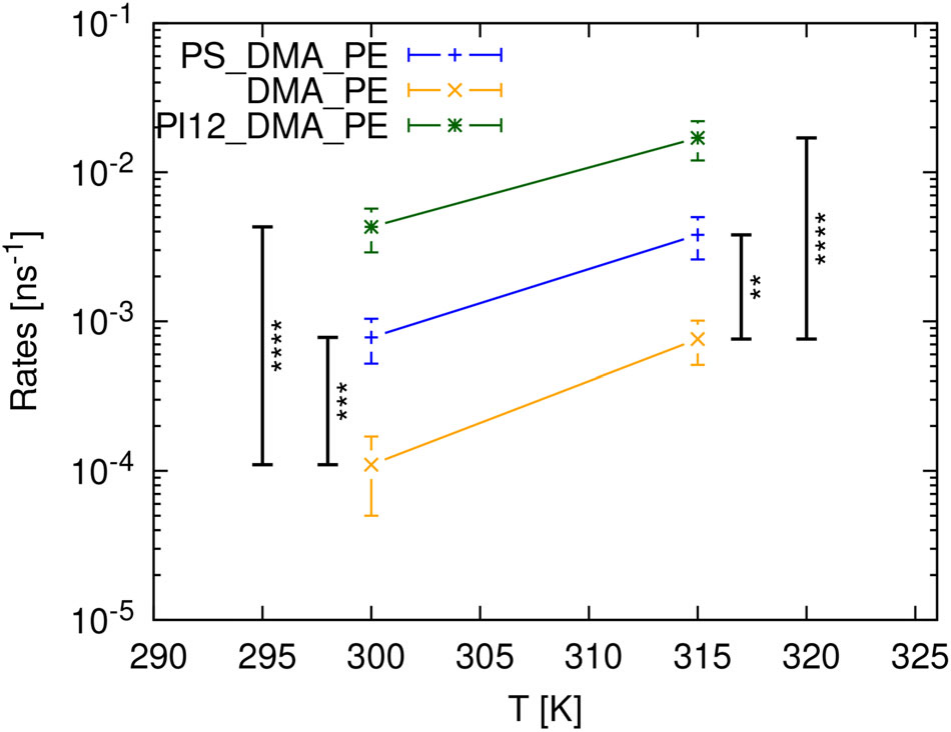
Average lamellar‐to‐hexagonal transition rates k of DODMA/DOPE (orange), DOPS/DODMA/DOPE (blue) and PI3P12/DODMA/DOPE (green) formulations as a function of temperature. The rates were computed as maximum likelihood estimate with right censoring from the data in Table 2 to include information from replicates where no transition happen [57]. The data reported in Table 2 are compatible with exponential distributions according to parametric bootstrap likelihood ratio tests (Weibull vs. exponential) [57]. The error bars are set to $k/\sqrt{n_{o}}$, where $n_{o}$ is the number of observed transitions. The *p*‐values of data comparisons (**, ***, and **** indicate *p*‐values ≤0.01, 0.001, and 0.0001, respectively) were obtained with Monte‐Carlo permutation log‐rank tests (10 000 permutations) [57].

The number of replicates in the lamellar phase decays exponentially with time, indicating that the transition is an activated process with a free energy of activation $\Delta G_{‡}$ that is linked to the average transition rate k through Eyring's equation $\Delta G_{‡}\propto \log k$. The data (Table 2, Figures 3a and 4) show that the transition times for the DODMA/DOPE mixtures are significantly larger (or equivalently that the transition rates are significantly smaller) than those for both the DOPS/DODMA/DOPE and PI3P12/DODMA/DOPE mixtures, indicating a smaller free energy barrier for the transition from lamellar to non‐lamellar phases in the ternary mixtures with lipid charge balance.

**Table 2:** List of times of onset in ns of the lamellar‐to‐hexagonal phase transition of each replicate run of the double bilayer simulations (simulation set 2).

	PS_DMA_PE_D^a^		DMA_PE_D^a^		PI12_DMA_PE_D^a^		PI25_DMA_PE_D^b^	
Replicate run	315 K	300 K	315 K	300 K	315 K	300 K	315 K	300 K
	Stalk formation time [ns]							
1	253.6	690.2	210.2	—	52.5	104.1	1736.8	—
2	1036.3	605.6	748.7	—	37.9	581.2	1893.3	—
3	200.6	932.8	1322.8	—	77.8	98.2	—	—
4	174.1	494.0	1771.8	2821.8	132.2	48.3	—	—
5	295.7	726.2	774.8	2686.4	23.2	496.8	—	—
6	53.5	1960.3	—	1253.8	11.9	417.4	—	—
7	4.6	1873.2	186.4	—	64.0	60.1	—	—
8	485.2	691.4	1577.8	—	127.2	99.4	—	—
9	75.8	556.4	913.5	—	25.0	39.6	—	—
10	39.9	—	1329.3	—	33.2	373.0	10074.9	—

^a^ Simulations extended to 3µs.

^b^ Simulations extended to 12µs.

Interestingly, the PI3P25/DODMA/DOPE mixture, where an equal amount of PI3P lipids (−3e charge) and DODMA lipids (+1e) are present, shows dramatically reduced transition rates suggesting that not any mixture of anionic and cationic lipids experiences a speed‐up of the transition. One possible reason for the lack of acceleration observed in the simulations could be represented by the presence of sodium counterions, necessary to neutralize the system, which may compete with the DODMA heads and reduce the probability of anionic–cationic lipid pair formation. This is supported by the density of ions and charged lipid beads along the direction orthogonal to the membrane (Figure S7), which shows that DODMA's head in PI3P25/DODMA/DOPE simulations is shifted deeper into the membrane than in PI3P12/DODMA/DOPE simulations by the presence of the sodium counterions. The reason why this phenomenon plays no role in the phase diagrams from simulation set 1 is related to the fact that the standard equilibration procedure used in set 1 simulations, which includes positional restraints on the heads of the lipids, helps pulling lipid heads together and reaching the transition state early.

It is worth noting that, as mentioned earlier, experiments support the occurrence of membrane charge neutralization after LNP fusion under physiological conditions [37], thus indicating that the PI3P25/DODMA/DOPE formulation, although helpful to understand the role of an excess of ions, may not represent the physiologically relevant situation.

Regarding the behavior of ions, sodium tends to penetrate slightly deeper in the lipid head layer than chlorine(Figure S5). This phenomenon is observed also in atomistic simulations, although the precise extent of it may vary from what observed in the present coarse‐grained simulations, keeping in mind that, in general, the treatment of ions both in atomistic and in coarse‐grained simulations is challenging [33]. Poisson‐Boltzmann expectations [56] (Figure S6) also predict larger sodium densities close to the phosphate groups; however, a continuum approximation may not be completely appropriate given the very small size of the water slabs and the discrete nature of the ions.

Assuming as mentioned above, that for the lamellar‐to‐hexagonal trasition rates k we have

(1) $$k\propto \exp\left(-\Delta G_{‡}/RT\right)=\exp\left(-\Delta H_{‡}/RT+\Delta S_{‡}/R\right)$$

with no other important temperature dependence (the prefactor to the exponential may be temperature dependent but it is assumed to provide only small corrections to the exponential dependence) and that $\Delta H_{‡}$ and $\Delta S_{‡}$, (the enthalpy and entropy change, respectively, between transition and lamellar state) are approximately constant in the range of analyzed temperatures, we can obtain $\Delta H_{‡}$ and $\Delta S_{‡}$ (Table 3) by fitting the measured rates in Figure 4. The errors are very large due to the availability of only two temperature points and a large correlation between the two parameters of the fit, thus it is hard to draw significant conclusions. However, the data may indicate that the lower transition rate for the DODMA/DOPE system compared to the ternary mixtures is possibly determined by a larger activation enthalpy (the sign of the activation entropy indicates that the entropy change would contribute to decrease the rates of the mixed systems, but the contribution at 315 K is smaller than the enthalpic one). As it will be shown in the next section, this would support the idea of the importance of the electrostatically driven anionic–cationic lipid pair formation at the transition state. It is important to note, however, that the transition rate of the lamellar‐to‐hexagonal transition provides information mostly on the free energy differences between the transition state and the lamellar state, while the only information that it returns about the hexagonal state is that it is more stable than the lamellar state. Only the rate of the hexagonal‐to‐lamellar transition could help inferring information on the enthalpic and entropic contribution to transition from the hexagonal state, but this “backward” rate is much lower than the “forward” rate that we have measured and out of the reach of our simulations. Thus, although the hexagonal phase is entropically more stable than the lamellar state, the speed of the lamellar‐to‐hexagonal transition may be controlled by an enthalpic stabilization of the transition state with respect to the lamellar state.

**Table 3:** Activation enthalpy and entropy for the lamellar‐to‐hexagonal phase transition obtained by fitting Eyring's equation to data from Figure 4.

Quantity	PS_DMA_PE_D	DMA_PE_D	PI12_DMA_PE_D
$\Delta H_{‡}$ (kJ/mol)	80 ± 24	99 ± 33	69 ± 23
$\Delta S_{‡}$ (J/mol/K)	−37 ± 78	8 ± 107	−59 ± 74

### 2.4 Transient Formation of Pairs of Anionic and Cationic Lipids

A structural characterization of the transition was obtained by analyzing the lipids involved in the stalk formation. Shortly, the stalk‐initiating lipids are those whose tails remain consistently parallel to the bilayer for a certain amount of time (the exact definition is provided and validated in the Methods section). The analysis shows that, in DOPS/DODMA/DOPE simulations, DOPS lipids are overrepresented in this set (33% mol. fraction) with respect to the 25% molar fraction in the whole simulation box. DODMA lipids are underrepresented (20% mol. fraction), instead. Similarly, in PI3P12/DODMA/DOPE formulations, PI3P is overrepresented in the stalk (62% mol. fraction vs. expected 12.5%), while DODMA (14% mol. fraction vs. 37.5%) and DOPE (24% mol. fraction vs. 50%) are underrepresented.

**Figure 5:**
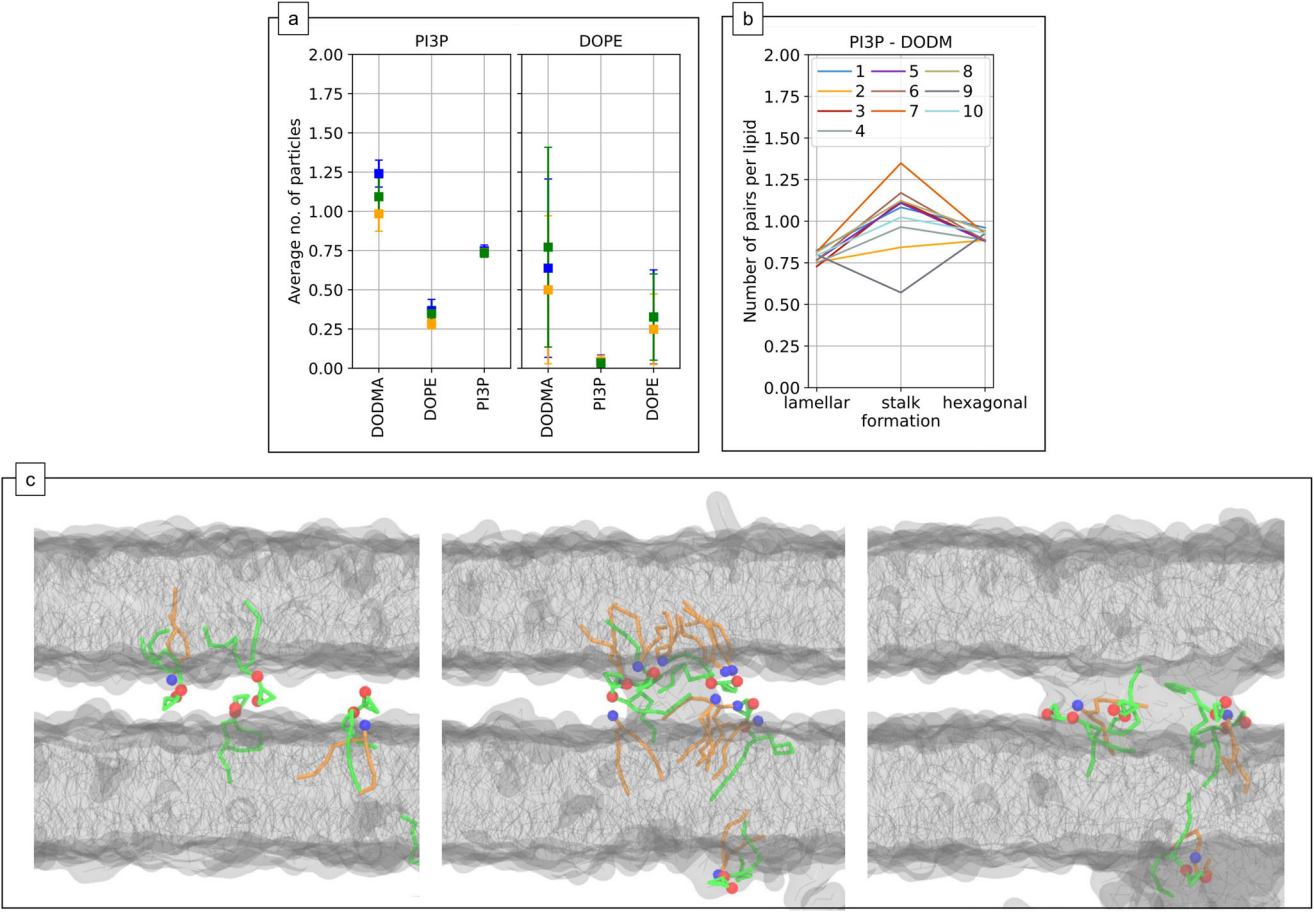
(a) Average number of charged head beads ($Q_{d}$ for DODMA, $Q_{a}$ for DOPE, and both $Q_{a}$ beads for PI3P) of the type reported on the *X* axis around the heads of stalk‐initiating lipids in the PI3P12/DODMA/DOPE (left for PI3P, right for DOPE) at 315 K and 2 w/l. Data from simulation set 2. The data is averaged over 20 ns windows in the lamellar phase (orange), during the stalk‐formation event (blue), and in the hexagonal phase (green), the standard deviation over the replicate runs is given as error bars. (b): Average number of DODMA lipids in contact with stalk‐initiating lipids in the PI3P12/DODMA/DOPE (data for stalk‐initiating PI3P). The stalk‐initiating lipids were identified at the stalk‐formation event, then, the same lipids were used to compute the number of contacting molecules in the lamellar and hexagonal phase, that is at the beginning and at the end of the simulation. (c) Snapshots of the stalk‐formation process in replicate run 1 of the PI3P12/DODMA/DOPE formulation at 315 K and 2 w/l. The carbon chains of all lipids are shown as gray thin lines and as enveloping surface. Stalk‐initiating PI3P molecules are shown in green with the phosphate group in red. DODMA molecules closer than 6.5 Å from the highlighted PI3P lipids are shown in orange with the methylamine group in blue. Water and non‐tail beads of all the other lipids are hidden for clarity. Snapshots are shown before (i) 33.2 ns and during (ii) 88.7 and (iii) 96.7 ns stalk formation (the stalk‐formation event starts at 77.8 ns).

The radial distribution functions (RDF) of the lipids were computed in a time window of 20 ns around the stalk‐formation event and integrated up to 0.65 nm (the location of the first minimum in the RDFs), which includes only the nearest lipid neighbors (Figure 3b). As terms of comparison, the equivalent integrals of the RDFs were also computed for the stretches of trajectory where the systems are in the lamellar phase and those, well after the transition, where the systems are in the hexagonal phase. The comparison shows that, for the DOPS/DODMA/DOPE system, at the onset of the transition the DOPS lipids in the stalk are in contact with a significantly larger number of DODMA lipids than in the other stages of the simulations (Figure 3b). A similar observation can be drawn analyzing the PI3P12/DODMA/DOPE system (Figure 5a).

This indicates that an excess of cationic–anionic lipid pairs transiently appear at the transition point with respect to the stable phases (Figures 3c,d and 5b,c). These pairs last only few nanoseconds (the average autocorrelation time of the lipid tail vectors is of the order of 1–2 ns), and are often already dissolved at the end of the 20 ns‐transition window (Figures 3d and 5c). In the DODMA/DOPE mixture no lipid exhibits a significantly increased tendency to take part to stalk formation and to form pairs (Figure 3b).

Counting the number of contacts made by stalk‐initiating lipids in the various runs provides a similar view (Figures 3c and 5b). Anionic lipids (DOPS or PI3P) lipids, when in the stalk, make in the vast majority of the cases more contacts with DODMA lipids than in the other phases. In the DODMA/DOPE simulation, DOPE lipids do not fully replace anionic lipids in pairing with DODMA at the stalk: an increase in the contacts at the stalk compared to other phases is observed only in a small fraction of the runs.

In combination with the reduction of transition times observed in the formulation mixing anionic and cationic lipids with respect to the DODMA/DOPE formulation, which indicates a reduction in the free energy barrier between the lamellar and hexagonal phases, the information provided above strongly supports the idea that it is the transient formation of pairs between cationic and anionic lipids, which induces the free energy barrier reduction.

## 3 Conclusion

The lipid formulations investigated in this work, comprising mixtures of ionizable cationic lipids alongside neutral and anionic phospholipids, as well as cholesterol, provide a simplified model of the endosomal membrane after a fusion event with a lipid‐based nanoparticle. The simulations recapitulate the anticipated destabilization of the lamellar phase in systems including both anionic and cationic lipids [7]. The characterization of the lamellar to hexagonal transition reveals that stalk formation acts as initiating event. Kinetic analysis shows that the presence of anionic and cationic species leads not only to a destabilization of the lamellar phase but also to a decrease of the free energy barrier separating the lamellar and the hexagonal phases, thereby accelerating the transition relative to formulations lacking one of the charged species. The observed phenomenon occurs with both the anionic lipids considered (DOPS and PI3P), notwithstanding the very different head structure, suggesting that, while specific lipid packing parameters and geometry may play a modulating role, anionic–cationic lipid pairing is the main determinant of the phenomenon.

A structural analysis of the stalk‐formation event shows a transient excess of contacts between anionic and cationic lipids relative to those observed in the stable phases. This suggests that the transition state for the lamellar to hexagonal transformation is stabilized by the presence of pairs of anionic and cationic lipids, which in turn determines the transition rate increase. The mechanistic insight provided by our study complements recent findings regarding the importance of pH‐dependent phase transitions in LNP formulations to determine transfection efficiency. In ref.  [8], it was shown that LNPs including lipids in the hexagonal phase improved transfection efficiency, while LNPs in the lamellar phase showed a delayed onset of the transfection due to the need to undergo first a transition to the hexagonal phase before endosomal escape, resulting in reduced transfection efficiency. Philipp et al. [9] showed that ionizable cationic lipids with a delayed endosomal release resulted in lower transfection efficiency. The delay was associated with the lower pH necessary to trigger the emergence of the hexagonal phase in bulk formulations including those lipids. Similar outcomes were reported in ref.  [10] where LNP formulations showing similarly high cell association resulted in different transfection efficiency depending on the pH of onset of the hexagonal phase, with higher pH resulting in larger transfection efficiency. Carucci et al.  [11] showed that buffers resulting in a raise of the pH of the $L_{2}$ to hexagonal phase transition in the analyzed formulation resulted in improved transfection efficiency.

The experiments reported above suggest that an early onset of the lamellar to hexagonal phase transition after fusion of the LNP with the endosomal membrane during endosomal maturation can result in improved transfection efficiency, indicating that the timing of the transition may be important. However, while in the experiments mentioned above the timing of the transition was achieved by acting only on the propensity of the LNP components (or buffer) to undergo the transition, our simulations suggest that the interactions with the anionic lipids of the endosomal membrane also have a significant impact on the speed of the transition and may, in principle, be exploited to improve transfection efficiency. In support of this argument, our data also provide a rationale for the observed enhancement in transfection efficiency upon incorporation of negatively charged lipids to standard LNP formulations [14].

Interestingly, multi‐tailed ionizable phospholipids, lipids [58] including a pH‐switchable zwitterionic headgroup made of a tertiary amine and a phosphate group, as well as three hydrophobic chains, mimicking the complexes resulting from the pairing of anionic and cationic lipids discussed here both in terms of shape and pH‐dependent behavior, have been shown to provide good transfection efficiency and, when combined with additional zwitterionic or cationic components, also selective organ targeting.

Although the results reported here support the idea that the lamellar‐to‐non‐lamellar phase transition conducive to successful endosomal escape is driven by anionic–cationic lipid interactions, they also show how helper lipids play an important modulating role. Changes in the concentration of gel‐phase‐forming helper lipids like DSPC in LNP formulations have been shown to affect the fusogenicity of the LNP by possibly changing the diffusion of lipids within the LNP, and altering the speed of structural rearrangements of lipids within the LNP, which is necessary for fusion [13, 46]. In the simulations we also observe that changes in the helper lipids composition (DOPE to cholesterol) have significant effects on the diffusion speed of lipids. Although these changes only partly affect the overall destabilization of the lamellar phase determined by the mixing of anionic and cationic lipids, they do significantly affect the speed of the transition, which may eventually affect, again, the timing of the event with respect to endosomal maturation and consequently alter the probability of a successful endosomal escape.

Furthermore, the composition of anionic lipids in the endosomal membrane varies as a function of cell type and stage of endosomal maturation [35], suggesting that by optimizing the interactions between cationic ionizable lipid and specific classes of anionic lipids could allow for precise control over the timing of endosomal escape and the tissue specificity of the delivery. Indeed, our simulations show that PI3P‐rich formulations undergo the lamellar‐to‐hexagonal transition faster than DOPS‐rich formulations when interacting with DODMA, suggesting that the interactions of the ionizable cationic lipid can be tuned to speed up the transition in the presence of specific anionic lipids.

The simulations presented here allowed for exposing the phenomenon of an acceleration of the lamellar‐to‐hexagonal phase transition induced by the interaction of oppositely charged lipids, which may have implications for the endosomal escape of the LNP cargo. While the coarse‐grained force field used here has allowed reaching the time scales necessary to observe the phenomenon, more accurate atomistic descriptions of the molecules will be required in future developments to either quantitatively analyze the commercially available ionizable cationic lipids or to help and design new ionizable lipids with improved interaction profiles. These descriptions may be built upon the present simulations by means of multi‐scale approaches [26, 30].

Overall, the findings reported here provide a mechanistic framework for the development of more effective lipid formulations, emphasizing the optimization of the interactions of ionizable cationic lipids and anionic lipids to enhance the efficacy and specificity of nucleic acid delivery systems.

## 4 Methods

The molecular models in the present simulations are based on the MARTINI representation [38, 39, 40, 41, 49]. For DOPE, DOPS, PI3P (PAP1 in the MARTINI lipidome nomenclature) and cholesterol the standard MARTINI 2.2 parametrization was used [59]. For the parametrization of DODMA, the MARTINI building block approach was applied and validated with atomistic simulations in previous works [26, 30] (Figure S9). As a model of the endosomal membrane after fusion with the LNP, the cationic ionizable lipids were considered charged (+1e) to reproduce the low pH environment in the endosome and the $Q_{d}$ bead was used to represent the charged head. The tails were modeled using the $C_{1}$ and $C_{3}$ beads to account for the presence of one unsaturated bond. The $N_{0}$ bead was used to model the ether group. The interaction matrix provided by MARTINI 2.2 with polarizable water was not changed. The program GROMACS 2020 [51, 52, 53, 54] was used to run the simulations of the single bilayer systems, while GROMACS 2023.3 [52, 53, 54, 60] was used to run the simulations of the double bilayer systems. For minimization and first equilibration, van der Waals interactions were cut off with potential‐shift‐verlet and the Coulomb interactions were treated with reaction field. In both cases, the cut‐off radius of 1.1 nm was used. The energy of the systems was minimized for 5000 step using a soft‐core potential to avoid singularities, followed by a further 5000 steps without soft‐core potential. Then, the systems were equilibrated at constant temperature and pressure following a five‐step procedure, each step corresponding to 1 ns equilibration run. The temperature of 270, 285, 300, or 315 K was controlled using the stochastic velocity rescaling [61] with a time constant of 1 ps. The pressure was kept at 1 atm with the Berendsen algorithm [62] using the semi‐isotropic pressure coupling with a time constant of 5 ps so that the axes along the bilayer plane were coupled. A restraining force was applied to the lipid heads whose force constant was reduced in each step from 200 to 100, 50, 20, and 10 kJ/mol/${\mathrm{nm}}^{2}$. At the same time, the time step was increased from 2 to 5, 10, 15 and 20 fs. The equilibration procedure follows ref. [63].

### 4.1 Simulation Set 1: Capturing the Phase Behavior

The equilibration was followed by a production run of 3 μs with a timestep of 20 fs. The same thermostat as for the equilibration was employed, while the Parrinello–Rahman algorithm [64] was used for the semi‐isotropic pressure coupling with a time constant of 12 ps. A cutoff of 1.2 nm for the van der Waals and the Coulomb interactions was set. For the long range electrostatic interactions, the Particle–Mesh–Ewald [65] method was applied. Four repeat runs were carried out for each combination of temperature, hydration level, and lipid formulation. The observed phases are consistent for all replicate runs. The runs showing a transition event to the inverted hexagonal phase were further elongated by additional 3 μs with anisotropic pressure coupling to let the sections of the water columns relax from an elliptical to a round shape. The same was applied to cases where a transition to an intermediate phase was observed. These simulations required a reduction of the timestep from 20 to 2 fs, to reduce unphysical deformations of the simulation box, possibly related to the GROMACS neighbor list implementation [66] although the corrections suggested in ref. [66] were applied (i.e., setting nstlist, nsttcouple, and nstpcouple to 20 steps while increasing rlist to 1.35 nm up to 2 nm and setting the verlet‐buffer‐tolerance to −1). Even so, no more than 0.5–2 μs physically reasonable trajectories could be collected before large box deformations were observed. Due to these instabilities emerging by using GROMACS, another set of runs starting from the intermediate phases at the end of the semi‐isotropic simulations was performed for 6 to 12 μs using OpenMM [55], with a Langevin thermostat [67] and the Monte Carlo anisotropic flexible barostat [68] to assess the stability of the phases. The other parameters of the simulations were kept the same as in the GROMACS runs.

### 4.2 Simulation Set 2: Transition Analysis

While simulation set 1 let us obtain the phase behavior over a large temperature and hydration range, we noticed that the free energy barrier for the transition is reduced by the restraints applied in the equilibration phase leading, in some case, to stalk formation already starting during the equilibration phase. For an in‐depth analysis of the kinetics, a more sophisticated setup was developed such that the transition takes place fully during the production run. To this extent, double bilayer systems were set up by doubling in the *z* direction the simulation box of the final frame of the corresponding single bilayer systems obtained after 3 μs of production run at 270 K and 2 water beads per lipid. In the case of the DOPS/DODMA/DOPE, the PI3P12/DODMA/DOPE, and the PI3P25/‐DODMA/DOPE systems, which did not show lamellar phase under these conditions, the final frame of the simulation with 3 water beads per lipid was used after gradually removing the excess water beads in steps. Each steps consisted of the removal of 10 water beads followed by 1 ns relaxation run at 315K. A total of 50 steps were necessary to reach the correct amount of water per lipid. On the double bilayer systems, 10 independent production runs of 3–12 μs for each formulation were carried out using the same protocol as above with the semi‐isotropic pressure coupling at temperatures of 300 and 315 K, resulting in 120 runs.

### 4.3 Simulation Set 3: Anisotropic Pressure Coupling for Water Column Re‐Assembly

The water columns in simulation set 2 are rotated by 30 degrees in the *xy*‐plane. Applying periodic boundary conditions, this leads to the insight that in reality one long column per layer has formed. This arrangement of the water columns has no consequences for most of the analysis. For the analysis of the angular distribution of the lipids around the columns; however, it was more convenient to have one long column without the need to reassemble it. Therefore, for the DOPS/DODMA/DOPE, the DODMA/DOPE, and the PI3P12/DODMA/‐DOPE formulations two simulations were run using GROMACS for 6 μs from the same starting frame as in simulation set 2 but with anisotropic pressure coupling and the Berendsen barostat using the corrections suggested in ref. [66] (see above). All other parameters remained the same as in simulation set 2. In the case of DOPS/DODMA/DOPE and PI3P12/DODMA/DOPE, both runs converged to one long water column. In the case of DODMA/DOPE, one single run reached the objective, while the second run underwent unphysical deformations of the simulation box of the same kind as discussed in ref. [66] and above.

### 4.4 Identification of the Stalk‐Formation Event and the Stalk‐Initiating Lipids

The first step in the lamellar‐to‐hexagonal phase transition is the formation of a stalk between two bilayer membranes. We have developed a procedure to identify the stalk‐formation event along the trajectory and the lipids initiating the event. To this extent, the trajectories have been scanned sequentially considering time windows of 20 ns with a step size of 0.1 ns. In each time window, after defining $z_{80}$ as the 20th percentile of the distribution of *z*‐projections of tail end‐to‐end vectors of the same lipid type within the window, all lipids have been marked whose (i) average *z*‐projections of both tails over the time window is less than $z_{80}$ Å (which implies that the lipid is consistently oriented parallel to the bilayer), (ii) the same projections measure less than $\left(z_{80}+0.5\right)$ Å for a consecutive stretch of trajectory of at least 1.5 ns; If in a time window at least five lipids are marked (criterion (iii)), the scanning is stopped, the stalk is considered as formed and the marked lipids are considered stalk‐initiating lipids. While it is not guaranteed that all lipids in the stalk are found with this method, all lipids identified do take part to the stalk. Small changes in the time window size, for example, a time window of 30 ns, do not significantly affect the results. To validate the choice of the method for identifying the stalk‐formation event, we have measured the commitment probability to stalk formation along the selected time windows (i.e., those windows where the stalk‐formation event has been located). We have selected trajectory frames along the time windows and have started, from each of them, 20 independent simulations of length 20 ns. The fraction of those simulation satisfying the above mentioned stalk‐formation criteria (i)–(iii) represents an estimate of the commitment probability of the starting frame. The data (Figure S10) show that the commitment probability increases along the time window, being close to 0 at the beginning and reaching values close to 0.5 (the transition state) within the time window, proving that the time window includes the transition state to stalk formation.

### 4.5 Average Number of Charged Head‐to‐Head Contacts

The average number of charged head beads ($Q_{d}$ for DODMA, $Q_{a}$ for DOPS, DOPE, and PI3P) around other charged head beads was obtained by integrating the number density of the heads up to the first minimum of the radial distribution function at 6.5 Å. Average and standard deviation are computed from the replicate runs of the double bilayer simulations (simulation set 2). The average number of beads is computed over a 20‐ns time window at the beginning of the trajectory, that is in the lamellar phase (orange), at the end of the trajectory, that is in the hexagonal phase (green) and at the stalk‐formation event (blue). The integrals are normalized to the number of lipids in the selection. The exact times of the stalk‐formation events in all the replicate runs are listed in Table 2.

### 4.6 Analysis

The lipids in all trajectories were made whole using the Gromacs utility *trjconv*. Plots were created using the python library Matplotlib [69] and gnuplot [70]. The normalized angular frequency of lipid heads as well as their xz‐distribution (Figure 1 de, the identification of the stalk‐initiating lipids (Table 2, Figure 3a, Section 4), the average number of charged head beads (Figure 3b), and the number of pairs per lipid (Figure 3c) were computed using our own python scripts and utilities of the python library MDAnalysis [71, 72] (lipid selection, distance matrix). For the tail order parameter (Figures S2 and S3), the MARTINI tool *do‐order* [73] was used. VMD was used for molecular pictures [74] (Figure 1, 2, 3, and 5c).

## Author Contributions

All authors conceived and initiated the project. D.N.Z. performed the simulations. D.N.Z. and G.S analyzed the data. All authors discussed the results, wrote and approved the final version of the manuscript.

## Conflicts of Interest

The authors declares no conflicts of interest.

## Supporting information


**Supporting File**: smll73434‐sup‐0001‐SuppMat.pdf.

## Data Availability

Simulation data and analysis scripts are made available on Zenodo (https://doi.org/10.5281/zenodo.15440147).
