# Supplementary material for: Mechanistic Insight Into Ionizable Cationic Lipid‐Mediated Endosomal Escape via Transient Lipid Complexes
Source: Small. 2026 Apr 26;22(32):e13399. doi: 10.1002/smll.202513399 (PMC13244417; doi:10.1002/smll.202513399)
Supplement: Supplementary file 1 — Supporting File: smll73434‐sup‐0001‐SuppMat.pdf. [file SMLL-22-e13399-s001.pdf]

Supplementary Information for: Mechanistic Insight into Ionizable Cationic Lipid–Mediated Endosomal Escape via Transient Lipid Complexes

David Noel Zimmer[1,2], Friederike Schmid[2], Giovanni Settanni[1,2*]

[1*]Faculty of Physics and Astronomy, Ruhr University Bochum, Universitaetsstrasse 150, Bochum, 44801, Germany.
[2]Institute of Physics, Johannes-Gutenberg University Mainz, Staudingerweg 9, Mainz, 55128, Germany.

*Corresponding author(s). E-mail(s): giovanni.settanni@rub.de;

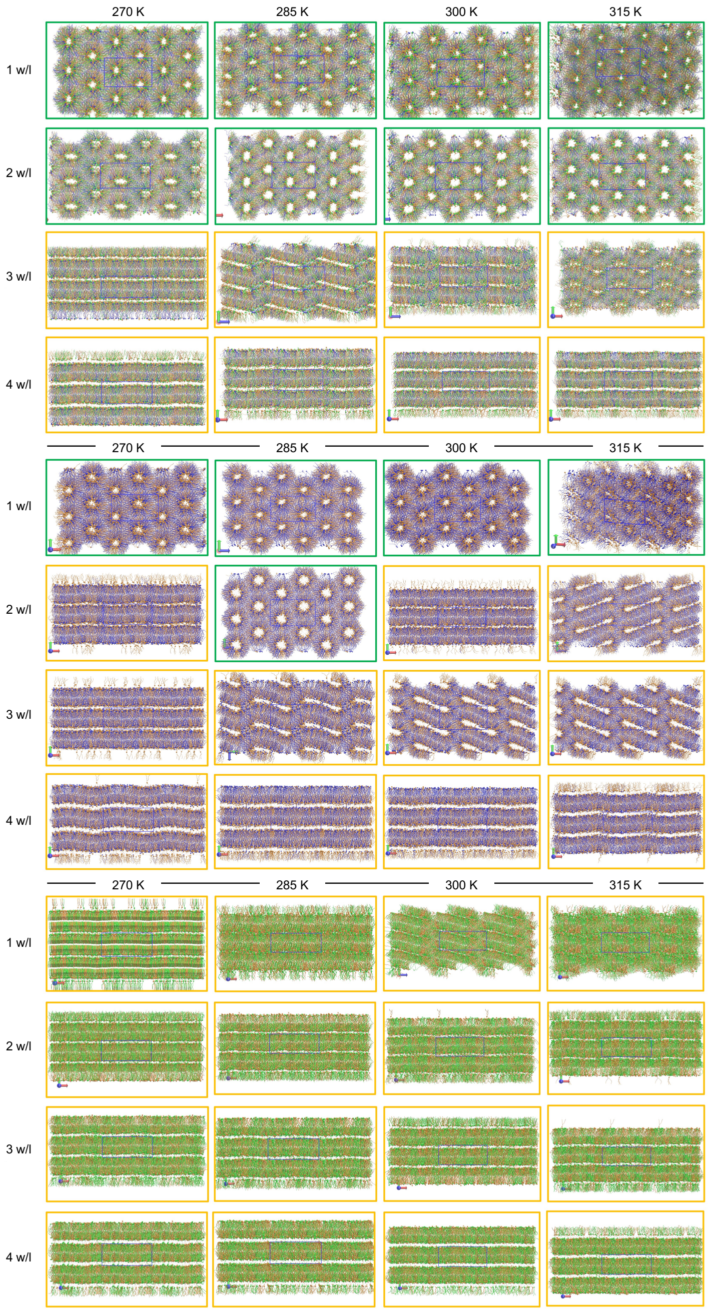

Fig. S1 Equilibrium structures of DOPS/DODMA/DOPE (a), DODMA/DOPE (b), and DOPS/-DOPE (c). DODMA is shown in blue, DOPS in green and DOPE in orange. Water was removed for clarity, the lipids are visualized using the licorice representation in VMD. The green frames around the box indicates simulations that have been extended using anisotropic pressure with GROMACS. Snapshots from simulation set 1.

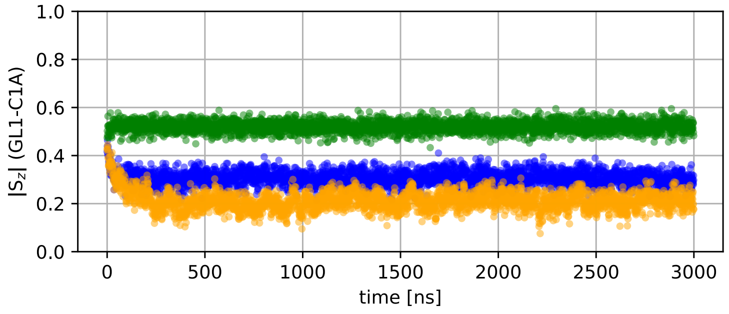

Fig. S2 Timeseries of the tail order parameter of the GL1-C1A bonds of DOPE at 315K and 2 w/l from the DOPS/DOPE formulation (green, lamellar phase), the DODMA/DOPE formulation (blue, intermediate phase) and the DOPS/DODMA/DOPE formulation (orange, hexagonal phase).

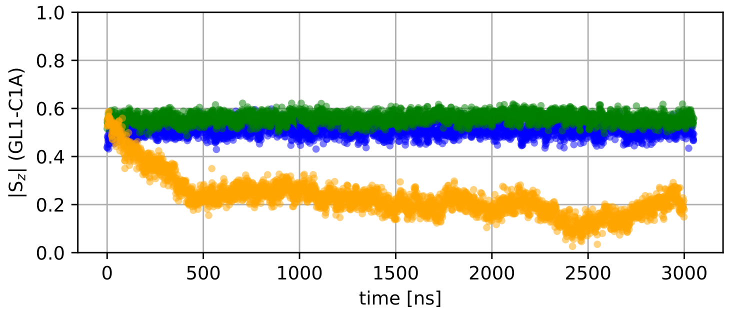

Fig. S3 Timeseries of the tail order parameter of the GL1-C1A bonds of DOPS (in the DOPS/-DODMA/CHOL: orange, hexagonal phase; in the DOPS/CHOL formulation: green, lamellar phase) and DODMA (in the DODMA/CHOL formulation: blue, lamellar phase) at 315K and 2 w/l.

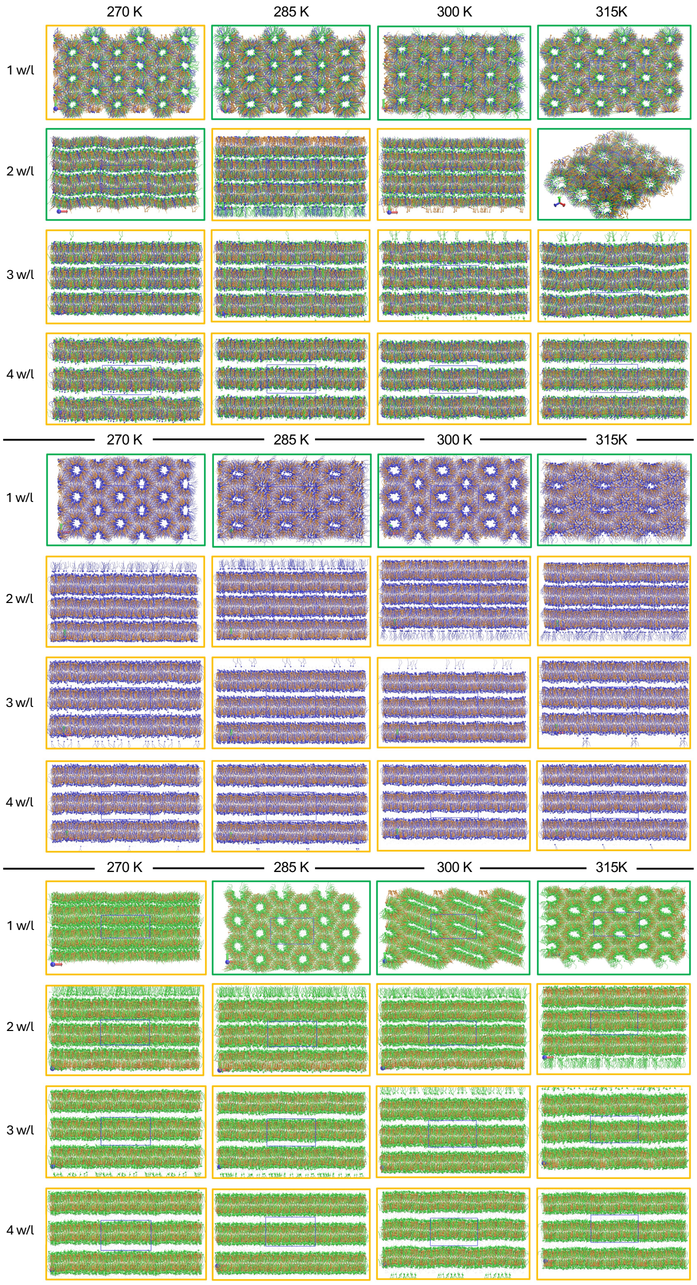

Fig. S4 Equilibrium structures of DOPS/DODMA/CHOL (a), DODMA/CHOL (b), and DOPS/-CHOL (c). DODMA is shown in blue, DOPS in green and CHOL in orange. Water was removed for clarity, the lipids are visualized using the licorice representation in VMD. The green frames around the box indicates simulations that have been extended using anisotropic pressure with GROMACS. Snapshots from simulation set 1.

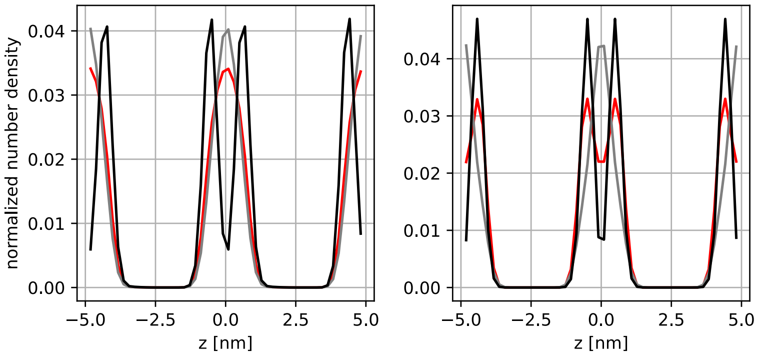

Fig. S5 The normalized densities along the z-direction of Cl- (left) and Na+ (right) shown in red, water shown in grey, and the charged head bead shown in black in DODMA/DOPE (left) and in DOPS/DOPE (right). Averaged over the replicate runs in simulation set 2 at 300 K.

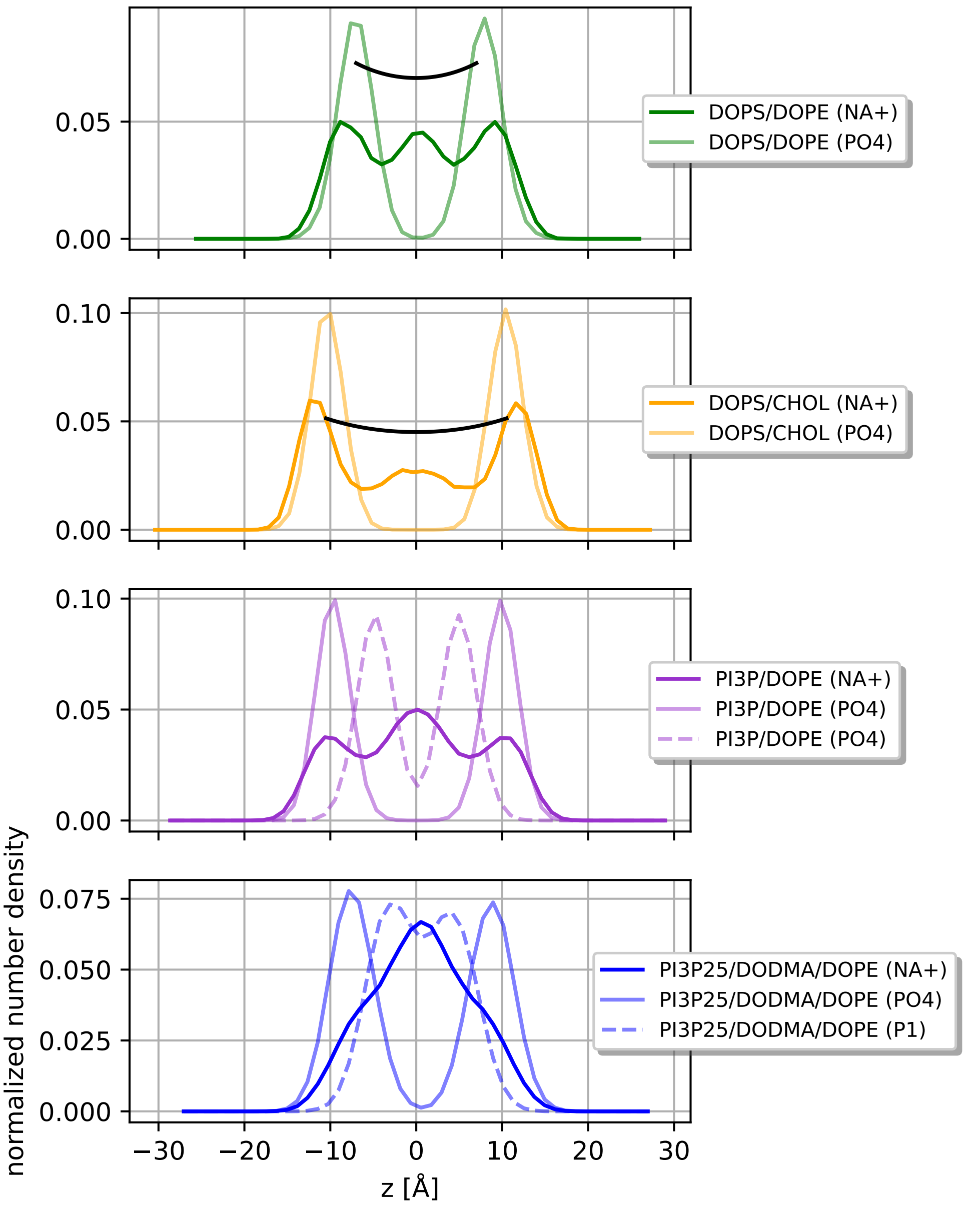

Fig. S6 The normalized densities along the z-direction of Na+ and the anionic head beads (PO4 main phosphate of phospholipids, P1 inositol-bound phosphate). Data are averaged over the replicate runs in simulation set 1 at 4 w/l and 300 K. The black lines correspond to the ion density predicted by the solution of the Poisson-Boltzmann equation in a water slab delimited by the position of the peak of the PO4 distribution. The small size of the water slab and the discrete nature of the ions highlight the inadequacy of the Poisson-Boltzmann approach in this case.

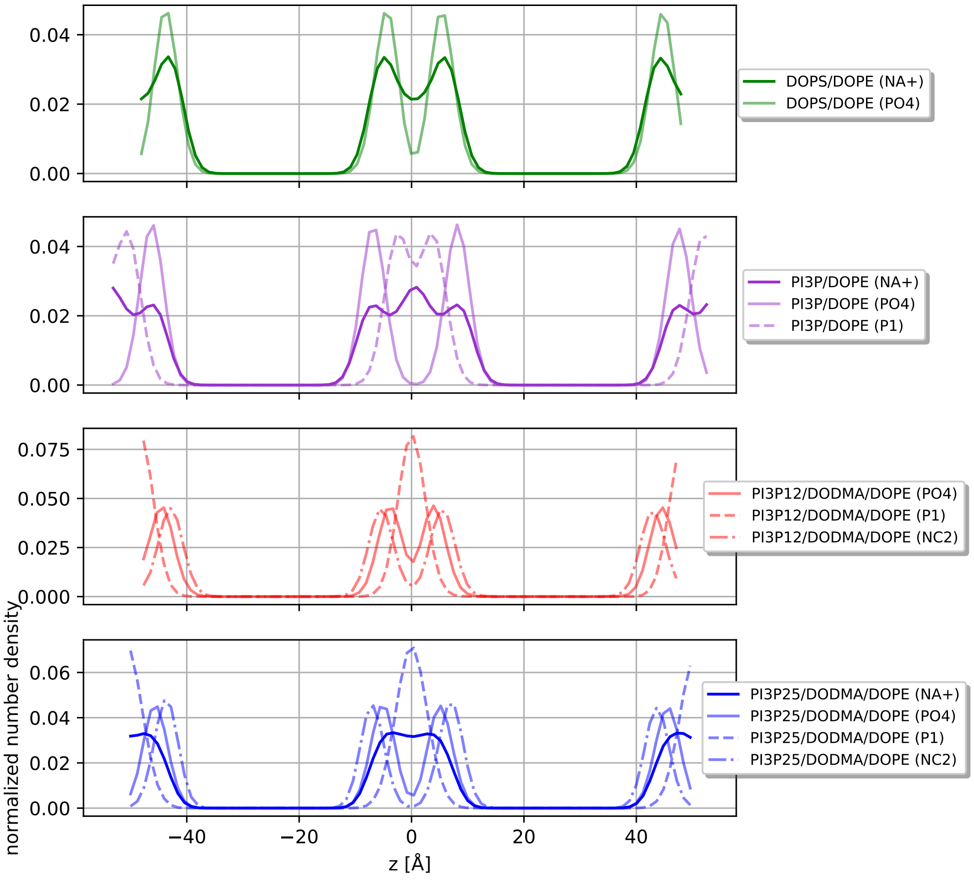

Fig. S7 The normalized densities along the z-direction of Na+ and the charged head beads averaged over the replicate runs in simulation set 2 at 2 w/l and 300 K. PO4 is the main phosphate group of phospholipids (charge -e), P1 is the inositol-bound phosphate of PI3P (charge -2e) and NC2 is the protonated head of DODMA (charge +e). The figure shows that sodium ions accumulate in the vicinity of PO4 and, in the simulations of PI3P25/DODMA/DOPE tend to push DODMA's head group deeper into the membrane, than in the PI3P12/DODMA/DOPE simulations where sodium is not present.

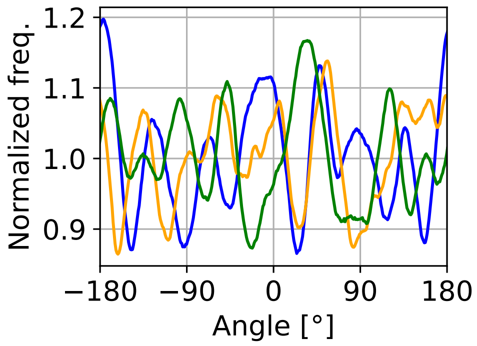

Fig. S8 Normalized angular frequency of observation of DOPS (green), DODMA (blue) and Cholesterol (orange) from the DOPS/DODMA/CHOL formulation around the water column formed in the hexagonal phase. Neighbor water columns are present in the directions at ±30, ±90 and ±150°. Schematic of the angle definition see Figure 1d.

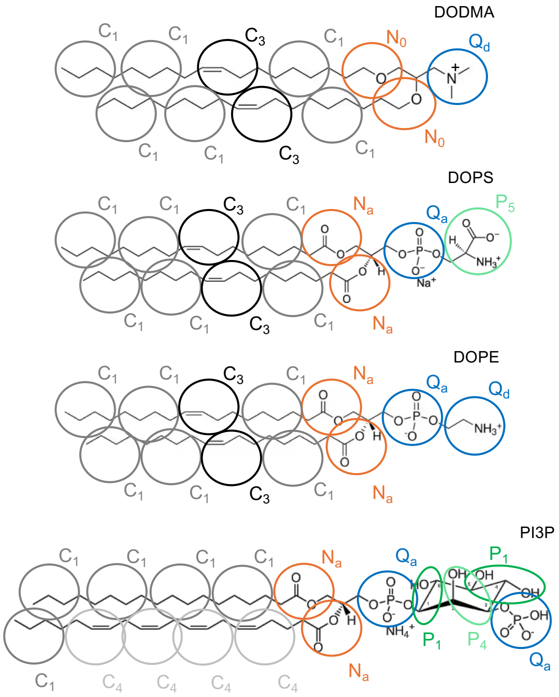

Fig. S9 Chemical structures of DODMA, DOPS, DOPE, and PI3P with the MARTINI bead representation superimposed.

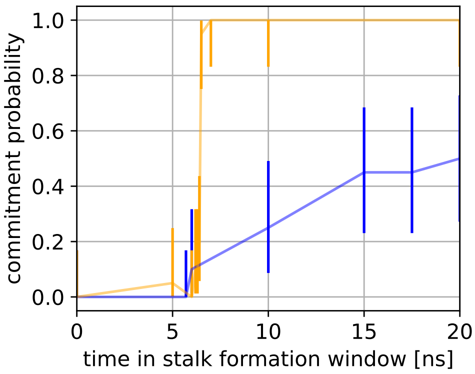

Fig. S10 The commitment probability to stalk formation for the DOPS/DODMA/DOPE formulation at 2 w/l and 315 K from simulation set 2 for replicate run 1 (blue) and 3 (orange) with error bars indicating the 95% confidence interval according to Clopper and Pearson[1].

Table S1 Area per lipid averaged over the repeat runs and the corresponding standard deviations. For simulation set 1, the replicate runs at 4 w/l and 300 K were used, for simulation set 2, the replicate runs at 2 w/l and 300 K were used. The last 1 µs was used for systems not undergoing a phase transition; otherwise, the time before stalk formation was used.

Formulation	Area per lipid [nm^2]
Simulation Set 1	
DOPS/DOPE	0.671 ± 0.001
DOPS/DODMA/DOPE	0.635 ± 0.001
DODMA/DOPE	0.632 ± 0.001
DOPS/CHOL	0.439 ± 0.001
DOPS/DODMA/CHOL	0.425 ± 0.001
DODMA/CHOL	0.441 ± 0.001
PI3P/DOPE	0.679 ± 0.001
PI3P12/DODMA/DOPE	0.635 ± 0.001
PI3P25/DODMA/DOPE	0.637 ± 0.001
Simulation Set 2	
DOPS/DOPE	0.628 ± 0.001
DOPS/DODMA/DOPE	0.588 ± 0.001
DODMA/DOPE	0.607 ± 0.001
PI3P/DOPE	0.648 ± 0.001
PI3P12/DODMA/DOPE	0.601 ± 0.001
PI3P25/DODMA/DOPE	0.618 ± 0.001

References

[1] Clopper, C. J. & Pearson, E. S. The use of confidence or fiducial limits illustrated in the case of the binomial. *Biometrika* **26**, 404–413 (1934). URL https://dx.doi.org/10.1093/biomet/26.4.404.
